# *NRG1* variant effects in patients with Hirschsprung disease

**DOI:** 10.1186/s12887-018-1265-x

**Published:** 2018-09-04

**Authors:** Gunadi, Nova Yuli Prasetyo Budi, Raman Sethi, Aditya Rifqi Fauzi, Alvin Santoso Kalim, Taufik Indrawan, Kristy Iskandar, Akhmad Makhmudi, Indra Adrianto, Lai Poh San

**Affiliations:** 1 Pediatric Surgery Division, Department of Surgery, Faculty of Medicine, Public Health and Nursing, Universitas Gadjah Mada/Dr. Sardjito Hospital, Yogyakarta, 55281 Indonesia; 2 Department of Pediatrics, Yoo Loo Lin School of Medicine, National University of Singapore, Singapore, 117549 Singapore; 3 The Khoo Teck Puat-National University Children’s Medical Institute, National University Hospital, Singapore, 119228 Singapore; 4 Department of Child Health, Faculty of Medicine, Public Health and Nursing, Universitas Gadjah Mada/UGM Academic Hospital, Yogyakarta, 55291 Indonesia; 5 Department of Public Health Sciences, Henry Ford Health System, Detroit, MI 48202 USA

**Keywords:** Hirschsprung disease, Indonesia, *NRG1* variant, Transcription factor binding motif

## Abstract

**Background:**

Hirschsprung disease (HSCR) is a heterogeneous genetic disorder characterized by absence of ganglion cells along the intestines resulting in functional bowel obstruction. Mutations in *neuregulin 1 (NRG1)* gene have been implicated in some cases of intestinal aganglionosis. This study aims to investigate the contribution of the *NRG1* gene to HSCR development in an Indonesian population.

**Methods:**

We analyzed the entire coding region of the *NRG1* gene in 54 histopathologically diagnosed HSCR patients.

**Results:**

All patients were sporadic non-syndromic HSCR with 53/54 (98%) short-segment and 1/54 (2%) long-segment patients. *NRG1* gene analysis identified one rare variant, c.397G > C (p.V133 L), and three common variants, rs7834206, rs3735774, and rs75155858. The p.V133 L variant was predicted to reside within a region of high mammalian conservation, overlapping with the promoter and enhancer histone marks of relevant tissues such as digestive and smooth muscle tissues and potentially altering the AP-4_2, BDP1_disc3, Egr-1_known1, Egr-1_known4, HEN1_2 transcription factor binding motifs. This p.V133 L variant was absent in 92 non-HSCR controls. Furthermore, the rs7834206 polymorphism was associated with HSCR by case–control analysis (*p* = 0.037).

**Conclusions:**

This study is the first report of a *NRG1* rare variant associated with HSCR patients of South-East Asian ancestry and provides further insights into the contribution of *NRG1* in the molecular genetic pathogenesis of HSCR.

**Electronic supplementary material:**

The online version of this article (10.1186/s12887-018-1265-x) contains supplementary material, which is available to authorized users.

## Background

Hirschsprung disease (HSCR), a heterogeneous genetic disorder, is characterized by the lack of ganglion cells along varying lengths of the intestines resulting in functional obstruction among children [1]. According to the length of aganglionosis, HSCR is categorized into three major types: short-segment (aganglionosis up to the upper sigmoid colon), long-segment (aganglionosis up to the splenic flexure and beyond) and total colonic aganglionosis (TCA) [1, 2].

The incidence of HSCR varies among populations with 15, 21 and 28 cases per 100,000 live births in Caucasians, Africans and Asians, respectively [1, 2]. These differences might be influenced by susceptibility factors such as the *REarranged during Transfection (RET)* rs2435357 risk allele frequency [3]. Our recent studies clearly demonstrated the presence of a higher frequency of *RET* rs2435357 susceptibility allele in Indonesian ancestry (50%) [4, 5]. Nevertheless, we observed that *semaphorin 3* rs11766001 common variant had different effects on HSCR depending on the ethnic background [6].

HSCR is a complex genetic disorder and at least 15 genes have been implicated in the development of HSCR, with the gene encoding the receptor tyrosine kinase *RET* accounting for up to 21% of sporadic cases [7]. These genes encode proteins that are important for the enteric ganglia development and are classified into three groups: 1) those that are associated with RET pathways (*RET, GFRα1, GDNF, NTN, PSPN*); 2) those implicated in endothelin type B receptor/EDNRB (*EDNRB, EDN3, ECE-1*) pathways; and 3) transcription factors that influence both RET and/or EDNRB pathways (*SOX10, ZFXH1B, PHOX2B*) [7, 8].

*Neuregulin 1 (NRG1)* has been implicated as a disease susceptibility gene in Chinese [9, 10]. Both common (single nucleotide polymorphisms, SNPs) and rare variants of this gene are reported to confer disease risk with over-representation in patient cohorts. However, in the first study on Caucasian, Luzón-Toro et al. [11] did not find any significant association of *NRG1* SNPs in Spanish HSCR suggesting that the association of such common polymorphisms to the disease may be restricted to specific populations. Furthermore, they found three novel *NRG1* rare variants that were hypothesized to have functional consequences during embryonic development of HSCR which could lead to HSCR in their patients. It is believed that *NRG1* plays an essential role in the signaling pathway of the enteric nervous system (ENS) [9–12]. In addition, the development of ENS requires a balance of neurogenesis and gliogenesis by RET/GDNF and ERBB2/NRG1 pathways, respectively. *Nrg1* suppresses *Gdnf*-induced neuronal differentiation and *Gdnf* negatively controls *Nrg1*-signaling by reducing the expression of its receptor, *ErbB2* [13]. Recently, we studied two previously reported *NRG1* SNPs and demonstrated that that only one common variant rs7835688 as a genetic risk factor for Indonesian HSCR [4]. Therefore, we aimed to perform a comprehensive study of the entire *NRG1* gene to investigate fully if there is any link between *NRG1* variants with Indonesian HSCR patients.

## Methods

### Patients

We recruited 54 patients with HSCR, of whom 38 were males and 16 females, corresponding to a gender ratio of 2.4:1. Diagnosis of HSCR at Dr. Sardjito Hospital, Yogyakarta, Indonesia was based on clinical findings, contrast enema and histopathology examinations. Hematoxillin-eosin staining and S100 immunohistochemistry were utilized for the histopathology assessment [4–6, 14–16]. For controls, 92 ethnically-matched individuals with no diagnosis of HSCR were enrolled.

The study was reviewed and approved by the Institutional Review Board (IRB) of the Faculty of Medicine, Universitas Gadjah Mada/Dr. Sardjito Hospital, Indonesia (KE/FK/787/EC/2015). Written informed consent was obtained from all parents of the subjects for this study.

### Genomic DNA extraction and polymerase chain reaction (PCR)

Genomic DNA was extracted from whole blood from each individual by using QIAamp DNA Extraction Kit (QIAGEN, Hilden, Germany), according to the manufacturer’s instructions. The extracted DNA samples were stored at − 20 °C until analysis.

PCR was conducted by using a Swift Maxi thermal cycler (Esco Micro Pte. Ltd., Singapore). The primer sequences for *NRG1* gene analysis were designed according to a previous study [10].

### DNA sequencing

To identify mutations, all 16 exons of the *NRG1* that had been PCR amplified in 20 fragments were screened using Sanger sequencing analysis with a BigDye Terminator V3.1 Cycle Sequencing Kit (Applied Biosystems, Foster City, CA). The products were separated and analyzed on a 3730xl Genetic Analyzer (Applied Biosystems, Foster City, CA) using DNA Sequencing Analysis Software (Applied Biosystems, Foster City, CA) [17, 18].

### Bioinformatics analyses

Data from the 1000 Genomes Project (http://www.internationalgenome.org) and ExAC (http://exac.broadinstitute.org) ancestry controls were utilized for comparison of variant frequencies among population. The in silico tools used to predict coding variant effects on protein function were SIFT (http://sift.jcvi.org/), PolyPhen-2 (http://genetics.bwh.harvard.edu/pph2/), LRT (https://www.ncbi.nlm.nih.gov/pmc/articles/PMC3910100/), Mutation Taster (http://www.mutationtaster.org), Mutation Assessor (http://mutationassessor.org/r3/), FATHMM (http://fathmm.biocompute.org.uk), CADD (http://cadd.gs.washington.edu) algorithm and DANN (https://cbcl.ics.uci.edu/public_data/DANN/) (Additional file 1).

SIFT predicts whether a change of amino acid change in a protein will have an impact on phenotype. This calculation is based on the premise that protein evolution is correlated with protein function, the more important positions of amino acids will correspondingly be more conserved in an alignment of the protein family. A SIFT score ranges from 0 to 1 and is classified as two groups: 1) predicted damaging if score ≤ 0.05; and 2) tolerated if score >  0.05 (http://sift.jcvi.org/). PolyPhen-2 score calculates the possible effect of a change of amino acid on the human protein structure and function. Its score ranges from 0 to 1.0 and consists of three interpretation: 1) benign (≤ 0.452 in PolyPhen2 HDiv and ≤ 0.446 in PolyPhen2 HVar); 2) possibly damaging (0.453 to 0.956 in PolyPhen2 HDiv and 0.447 to 0.909 in PolyPhen2 HVar); and 3) probably damaging (≥ 0.957 in PolyPhen2 HDiv and ≥ 0.909 in PolyPhen2 HVar) (http://genetics.bwh.harvard.edu/pph2/). Mutation Taster determines the disease potential of an alteration of amino acid using the Bayes classifier, with four possible analysis: 1) disease causing (i.e. probably deleterious); 2) disease causing automatic (i.e. known to be deleterious); 3) polymorphism (i.e. probably harmless); and 4) polymorphism automatic (i.e. known to be harmless) (http://www.mutationtaster.org). DANN uses deep neural network (DNN) algorithm to predict pathogenicity of variants and scores from 0.98 to 1 are considered to be protein disrupting (Additional file 1). All these tools predict whether a mutation leads to damaging function that may be potentially disease causing but it is well acknowledged that such in silico tools are only used as predictive guides and may not necessarily agree with each other as they can yield conflicting results [19–21].

The predicted conservation scores of variants were determined using GERP (http://mendel.stanford.edu/SidowLab/downloads/gerp/index.html), PhyloP (http://ccg.vital-it.ch/mga/hg19/phylop/phylop.html), and SiPhy (http://portals.broadinstitute.org/genome_bio/siphy/index.html) tests. The clinical significance of variants was analyzed using ClinVar tool (https://www.ncbi.nlm.nih.gov/clinvar/). The HaploReg database (https://pubs.broadinstitute.org/mammals/haploreg/haploreg.php) was utilized to evaluate the impact of the variant by annotation of epigenomic, conservation and regulatory motif information (Additional file 1).

### Statistical genetic analysis

Chi-square test was used to establish *p*-values for the case–control association analysis for *NRG1* variants (rs7834206, rs3735774, and rs75155858). A *p*-value less than 0.05 was considered significant.

## Results

All fifty-four patients (*n* = 54) were sporadic non-syndromic HSCR. Neither familial nor syndromic HSCR were included in this study. According to the degree of aganglionosis, the distribution of the cases was 53/54 (98%) short-segment, 1/54 (2%) long-segment and none were TCA. The mean age at diagnosis and age at definitive operation was 34.6 ± 44.5 months (range, 1–174 months) and 38.7 ± 43.9 months (range, 1–175 months), respectively (Table 1).

**Table 1:** Clinical characteristics of Indonesian HSCR patients

Characteristics	N (%); months
Gender	
Male	38 (70)
Female	16 (30)
Age at diagnosis	34.6 ± 44.5
Degree of aganglionosis	
Short segment	53 (98)
Long segment	1 (2)
Age at definitive surgery	38.7 ± 43.9
Type of surgical approach	
Transanal endorectal pull-through	21 (43)
Duhamel procedure	12 (25)
Soave procedure	11 (22)
Others	5 (10)

*HSCR* Hirschsprung disease

**Fig. 1:**
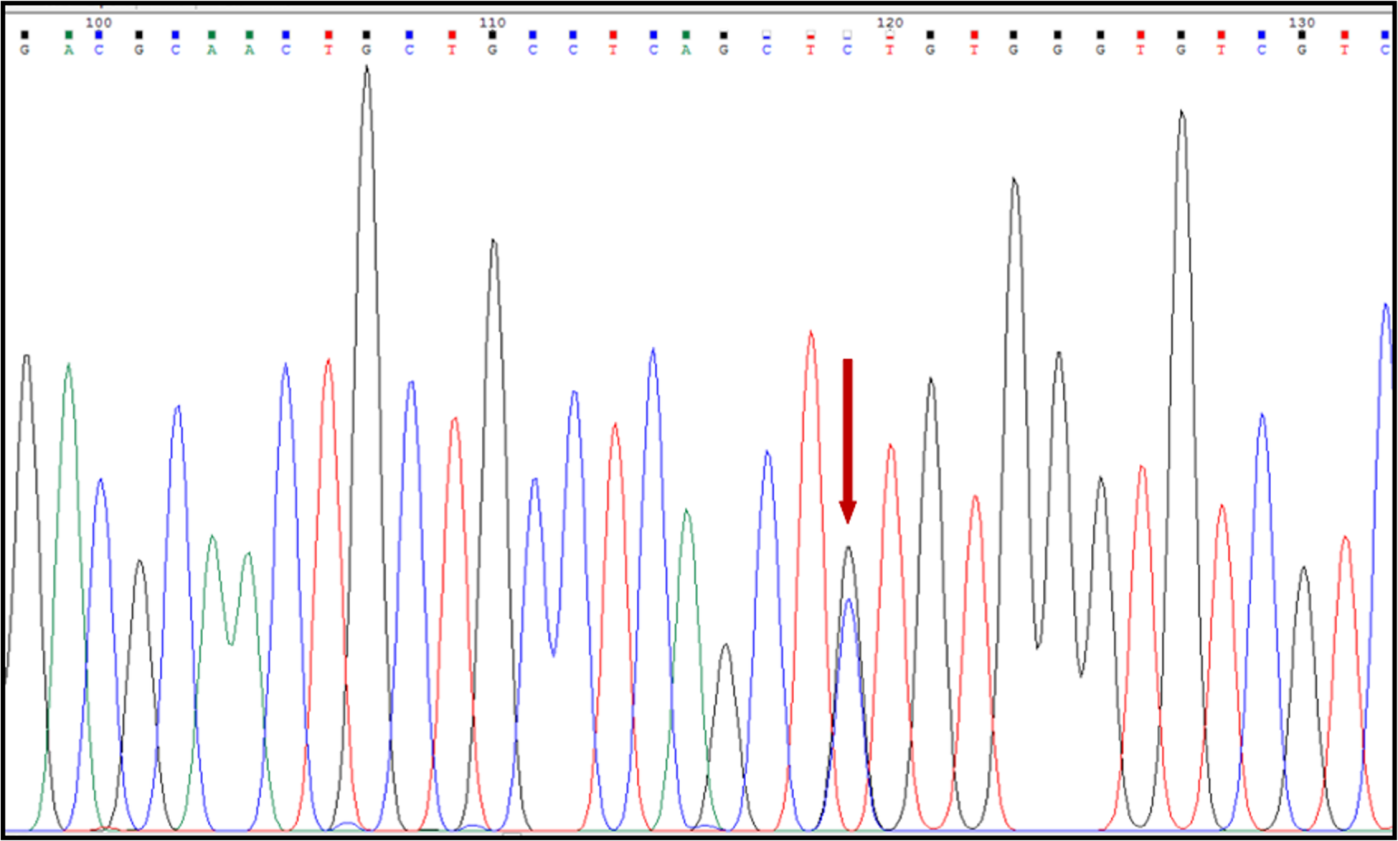
Sanger sequencing of *NRG1* exon 7 showed a missense variant, c.397G > C, which led to substitution of valine with leucine at amino acid 133 (p.V133 L) in the NRG1 protein. The arrow indicates the mutation

DNA analysis covering coding regions of all 16 exons of *NRG1* gene in our cohort of 54 Indonesian HSCR patients demonstrated the presence of four variants. These variants were: one rare missense variant, c.397G > C, in exon 7, which led to a substitution of valine with leucine (p.V133 L) in one HSCR patient (Fig. 1), and three common variants, rs7834206, rs3735774, and rs75155858 (Table 2).

**Table 2:** *NRG1* variants found in Indonesian HSCR patients

Variants		Reference	Frequency		Odds ratio (95% CI)	*p*-value
Nucleotide	Amino Acid		Cases/Allele (54/108)	Control/Allele (92/184)		
c.-97C > A	5’-UTR	rs7834206	Genotype	Genotype		
			CC: 26	CC: 59	1.79 (1.03–3.11)	0.037*
			CA: 24	CA: 31		
			AA: 4	AA: 2		
			Allele	Allele		
			C: 76	C: 149		
			A: 32	A: 35		
c.136G > A	p.G46R	rs3735774^α^	Genotype	Genotype		
			GG: 47	GG: 75	0.79 (0.33–1.89)	0.59
			GA: 6	GA: 17		
			AA: 1	AA: 0		
			Allele	Allele		
			G: 100	G: 167		
			A: 8	A: 17		
c.2298G > T	p.G613 V	rs75155858^μ^	Genotype	Genotype		
			GG: 26	GG: 39	1.02 (0.62–1.67)	0.94
			GT: 18	GT: 42		
			TT: 10	TT: 11		
			Allele	Allele		
			G: 70	G: 120		
			T: 38	T: 64		
c.397G > C	p.V133 L	rs35641374^α^	Genotype	Genotype		
			GG: 53	GG: 92		
			GC: 1	GC: 0		
			CC: 0	CC: 0		
			Allele	Allele		
			G: 107	G: 184		
			C: 1	C: 0		

^*^, a *p*-value of < 0.05 was considered significant; ^α^, NP_039253; ^μ^, NP_039251

We compared the observed *NRG1* rare variant allele frequency in Indonesian HSCR patients with those reported for the 1000 Genomes Project and ExAC ancestry controls. The frequency of c.397G > C (p.V133 L) rare variant in our HSCR patients (0.9%) was higher than those reported for the 1000 Genomes and ExAC ancestry controls (0 and 0.07%, respectively, *p* < 0.0001) (Table 3).

**Table 3:** *NRG1* variants frequency in Indonesian HSCR and population databases

Variant	HSCR patients	Indonesian control	1000 Genomes^μ^	ExAC^μ^	*p*-value	
					vs. 1000 Genomes	vs. ExAC
c.397G > C (p.V133 L)	0.009	0	0	0.0007	< 0.0001	< 0.0001
rs7834206	0.30	0.19	0.18	N/A	< 0.0001	N/A
rs3735774	0.07	0.09	0.09	0.07	1	1
rs75155858	0.35	0.35	0.35	0.35	1	1

*, a *p*-value of < 0.05 was considered significant; ^μ^, East Asian ancestries; HSCR, Hirschsprung disease; N/A, not available

Next, we analyzed the potential damaging effect of the rare variant (p.V133 L) using the following function prediction algorithms: SIFT, PolyPhen-2 (HDiv and HVar), LRT, Mutation Taster, Mutation Assessor, FATHMM, CADD and DANN (Table 4). Although SIFT and PolyPhen-2 analysis of p.V133 L showed the variant as being tolerated (0.22) and benign (0.029 in PolyPhen2 HDiv and 0.02 in PolyPhen2 HVar), respectively, p.V133 L was predicted to be disease causing by Mutation Taster and DANN. According to the conservation scores prediction using GERP and PhyloP vertebrate, the p.V133 L variant reached a deleterious threshold with a score of 4.26 and 1.799, respectively (Table 5) [22, 23]. However, ClinVar reported the clinical significance of the p.V133 L variant as likely benign (Table 6).

**Table 4:** Prediction of *NRG1* variants effects on protein function

Variant	SIFT	Polyphen2 – HDIV	Polyphen2 – HVAR	LRT	Mutation Taster	Mutation Assessor	FATHMM	CADD	DANN
c.397G > C (p.V133 L)	0.22 (tolerated)	0.029 (benign)	0.02 (benign)	0 (neutral)	Disease causing	0.805 (low)	0 (tolerated)	12.29 (benign)	0.9892 (protein disrupting)
rs7834206	0	0	0	0	0	0	0	0	0.6874 (non-protein disrupting)
rs3735774	0.36 (tolerated)	0.003 (benign)	0.004 (benign)	0	Polymorphism	0.345 (neutral)	0	8.83 (benign)	0.9957 (protein disrupting)
rs75155858	0	0.962 (probably damaging)	0.784 (possibly damaging)	0.004 (neutral)	Polymorphism automatic	0.895 (low)	0.4 (tolerated)	14.6 (benign)	0.9934 (protein disrupting)
Prediction scores interpretation:									
Method				Deleterious cut-off					
SIFT				< 0.05					
Polyphen2 HDIV				> 0.453					
Polyphen2 HVAR				> 0.447					
LRT				> 0.999					
Mutation Taster				D (disease causing) or P (polymorphism)					
Mutation Assessor				> 0.65					
FATHMM				< −1.5					
CADD				> 15					
DANN				> 0.98 (protein disrupting) 0.93–0.98 (splice site/promoter region) < 0.93 (non-protein disrupting)

**Table 5:** Conservation scores of *NRG1* variants

Variant	GERP	PhyloP placental	PhyloP vertebrate	SiPhy
c.397G > C (p.V133 L)	4.26 (deleterious)	1.55 (non-deleterious)	1.799 (deleterious)	10.121 (non-deleterious)
rs7834206	0	0	0	0
rs3735774	5.15	2.894	0.784	9.461
rs75155858	4.71	1.321	3.472	10.265
Conservation scores interpretation:				
Method		Deleterious cutoff		
GERP		> 2		
PhyloP		> 1.6		
SiPhy		> 12.17

**Table 6:** Clinical significance of *NRG1* variants

Variant	Clinical Significance	ClinVar ID
c.397G > C (p.V133 L)	Likely benign	RCV000202884.1
rs7834206	0	0
rs3735774	0	0
rs75155858	0	0

We then used the HaploReg database to assess the regulatory potential of this variant. This variant was predicted to reside within a region of high mammalian conservation, overlapping with the promoter and enhancer histone marks of relevant tissues such as digestive and smooth muscle tissues and altering AP-4_2, BDP1_disc3, Egr-1_known1, Egr-1_known4, HEN1_2 transcription factor binding motifs (Table 7). When 92 Indonesian control subjects were screened, none carried this variant, suggesting that it is not likely to be a common variant in our population.

**Table 7:** HaploReg v4.1 bioinformatics database search of *NRG1* p.V133 L

Variant	Histone H3K4me1 (Enhancer)	Histone H3K4me3 (Promoter)	Histone H3K9ac (Promoter)	Motifs Change
c.397G > C (p.V133 L)	Gastric	Duodenum Smooth Muscle, Colon Smooth Muscle, Stomach Smooth Muscle, Fetal Stomach, Fetal Intestine Small, Fetal Intestine Large	Stomach Mucosa	AP-4_2, BDP1_disc3, Egr-1_known1, Egr-1_known4, HEN1_2

Next, we compared the risk allele frequencies of the three *NRG1* common variants in 54 Indonesian HSCR cases and 92 Indonesian controls (Table 2). For rs7834206, the risk allele (A) has a frequency of 29.6% (32/108) in cases as compared to 19% (35/184) in controls, and the frequency in patients is 1.79-fold higher and significantly so (*p* = 0.037). The risk allele frequencies of rs3735774 (allele A) and rs75155858 (allele T) are similar in cases and controls [allele frequency in cases: 7.4% (8/108) and 35.2% (38/108); in controls: 9.2% (17/184) and 34.8% (64/184), respectively, (with *p* = 0.59 and 0.94), respectively] (Table 2). The genotypes of *NRG1* rs7834206, rs3735774, and rs75155858 were in Hardy–Weinberg equilibrium with the *p*-values of 0.37, 0.59, and 0.94, respectively.

Furthermore, we checked the rare and common variants from previous studies [10, 11] in Indonesian HSCR patients. We could not detect any rare variant found in previous studies [10, 11] in our cohort of HSCR patients, and the following rare variants [10] have been now considered as common variants: p.H347Y (rs758262997), p.P356L (rs776232660), p.A511T (rs376858256), and p.P608A (rs201432506) (http://www.internationalgenome.org).

## Discussion

In this study, we have conducted a mutational screening of the *NRG1* gene in an Indonesian cohort of HSCR patients. We found three non-synonymous variants and one nucleotide substitution variant in the 5′ untranslated region of the *NRG1* gene. Among them, p.V133 L located in the EGF-like domain within the NRG1 protein, was considered a rare variant in our population since it was absent in 92 non-HSCR controls. There was conflicting results from computational prediction programs with regards to the pathogenicity of p.V133 L and it has been classified as benign in ClinVar. However, the location of this variant in a well conserved and important histone and motif binding region suggests that it may have a regulatory function on gene expression.

The EGF domain where p.V133 L resides has been shown to be necessary for the activation of ErbB receptors [24]. The variant might also affect the *SOX10*-mediated maintenance of ENS progenitors since the receptors for *Nrg1* and *ErbB3* are regulated by *Sox10* [25, 26]*.* Moreover, the ENS development involves a balance of neurogenesis and gliogenesis by RET/GDNF and ERBB2/NRG1 pathways, respectively [13]. Therefore, we might suggest that p.V133 L will have an effect in intestinal aganglionosis in our HSCR patient by two mechanisms: 1) affecting the *SOX10*-mediated maintenance of ENS progenitors, and 2) altering the balance of neurogenesis and gliogenesis during ENS development.

The p.V133 L variant was absent in 92 non-HSCR population. Thus, we considered the variant as rare in our population. The presence of this variant was also higher in our patients than those reported in the 1000 Genomes and ExAC ancestry controls (0.9% vs. 0 vs. 0.07%). Previous studies reported *NRG1* rare variants to be implicated in the development of ENS and HSCR [10, 11]. Interestingly, the frequency of this *NRG1* rare variant found in Indonesian [1/54 (1.8%) patients] was similar to those of Caucasian and Chinese probands [3/207 (1.4%) and 13/358 (3.6%) patients, respectively; with *p* = 0.45] [10, 11]. Furthermore, the *NRG1* rs7835688 common variant was originally associated with HSCR in Chinese patients [9] and has been similarly observed in other Asian ancestry cases [4, 27], but these associations were not replicated in any Caucasian population [11, 28].

We were able to find evidence of the genetic effect of *NRG1* rs7834206 in Indonesian HSCR cases. Our study shows that *NRG1* rs7834206 is a genetic risk factor for HSCR with a background allele frequency of ~ 19% in Indonesian populations (Table 3). The risk allele at rs7834206 (0.19 vs. 0.18) in Indonesian controls has a similar frequency to subjects of Asian ancestry in the 1000 Genomes dataset (*p* = 0.80). Furthermore, a recent meta-analysis study showed that *NRG1* polymorphisms are risk factors for HSCR in Asians but not in Caucasians [29]. Therefore, our findings strengthen the notion of the potential damaging role of *NRG1* common variants in HSCR patients of Asian ancestries. It should be noted that the small sample size in this study poses a potential limitation and a significantly larger number of patients are needed to validate these observations. Future investigations on larger cohorts will clarify the exact role of the two variants, rs7834206 and p.V133 L in HSCR in Indonesian population. Moreover, moving beyond the prediction effect of mutations, further in vitro or in vivo functional studies are required to shed light on the actual effect of any variant.

Although there have been previous studies correlating *NRG1* SNPs with disease association in HSCR, these have only been reported in three populations, namely Chinese, Caucasian, and Thais [10, 11, 27, 28]. Indeed, contrary to the findings in Chinese, Luzón-Toro et al. [11] failed to find association of *NRG1* variants with HSCR phenotype in Spanish patients and suggested that this could be due to population differences. In the Thai patients, only four *NRG1* SNPs were selected for association studies and disease correlation was found in Thai-Chinese but not Thai-Muslim patients suggesting ethnicity differences [27]. Our present study is unique in this being a comprehensive screen of *NRG1* gene in Indonesian HSCR patients. As indicated in all the above studies, it is important to verify the role of *NRG1* variants in different population groups due to ethnicity differences. Furthermore, the SNPs in this study, namely the three common variants (rs7834206, rs3735774, and rs75155858) have not been previously investigated in all the other reported studies [10–12]. Since the allele frequencies of some common variants have been known to vary among different ethnic groups within Asian population, it is thus important to clarify the relationship of *NGR1* SNPs in Indonesian patients [30].

In view of recent interest in the role of *NRG1* in HSCR, it is not surprising that there is increasing evidence showing the contributions of both common and rare variants of this gene to disease risk. Our study reinforces this growing body of information showing the presence of *NRG1* risk variants that may interact with other alleles such as *RET, GDNF, ErbB or SOX10* as well as other susceptibility factors in leading to HSCR risk, and demonstrating that there are population differences in the contributions of the different risk alleles.

## Conclusion

This study is the first report of a *NRG1* rare variant associated with HSCR patients in South-East Asian ancestry and adds insights into the role of *NRG1* in the molecular pathogenesis of HSCR.

## Additional file

Additional file 1: Supplementary material URLs. (DOC 22 kb)

## Abbreviations

ENS: Enteric nervous system
HSCR: Hirschsprung disease
NRG1: Neuregulin 1
PCR: Polymerase chain reaction
RET: REarranged during Transfection
SNPs: Single nucleotide polymorphisms
TCA: Total colonic aganglionosis

## References

[1] Chakravarti A, Lyonnet S, Scriver CR, Beaudet AL, Valle D (2001). Hirschsprung disease. The metabolic and molecular bases of inherited disease.

[2] Amiel J, Sproat-Emison E, Garcia-Barcelo M (2008). Hirschsprung disease, associated syndromes and genetics: a review. J Med Genet.

[3] Emison ES, Garcia-Barcelo M, Grice EA (2010). Differential contributions of rare and common, coding and noncoding ret mutations to multifactorial Hirschsprung disease liability. Am J Hum Genet.

[4] Gunadi KA, Ling AY (2014). Effects of RET and NRG1 polymorphisms in Indonesian patients with Hirschsprung disease. J Pediatr Surg.

[5] Gunadi, Dwihantoro A, Iskandar K, Makhmudi A, Rochadi (2016). Accuracy of polymerase chain reaction-restriction fragment length polymorphism for RET rs2435357 genotyping as Hirschsprung risk. J Surg Res.

[6] Gunadi, Makhmudi A, Agustriani N, Rochadi (2016). Effects of SEMA3 polymorphisms in Hirschsprung disease patients. Pediatr Surg Int.

[7] Alves MM, Sribudiani Y, Brouwer RW (2013). Contribution of rare and common variants determine complex diseases-Hirschsprung disease as a model. Dev Biol.

[8] Tam PK, Garcia-Barceló M (2009). Genetic basis of Hirschsprung's disease. Pediatr Surg Int.

[9] Garcia-Barcelo MM, Tang CS, Ngan ES (2009). Genome-wide association study identifies NRG1 as a susceptibility locus for Hirschsprung's disease. Proc Natl Acad Sci U S A.

[10] Tang CS, Ngan ES, Tang WK (2012). Mutations in the NRG1 gene are associated with Hirschsprung disease. Hum Genet.

[11] Luzón-Toro B, Torroglosa A, Núñez-Torres R (2012). Comprehensive analysis of NRG1 common and rare variants in Hirschsprung patients. PLoS One.

[12] Yang D, Yang J, Li S (2017). Effects of RET, NRG1 and NRG3 polymorphisms in a Chinese population with Hirschsprung disease. Sci Rep.

[13] Gui H, Tang WK, So MT (2013). RET and NRG1 interplay in Hirschsprung disease. Hum Genet.

[14] Setiadi JA, Dwihantoro A, Iskandar K, Heriyanto DS (2017). Gunadi. The utility of the hematoxylin and eosin staining in patients with suspected Hirschsprung disease. BMC Surg.

[15] Parahita IG, Makhmudi A, Gunadi. Comparison of Hirschsprung-associated enterocolitis following soave and Duhamel procedures. J Pediatr Surg. 2018;53:1351-135. 10.1016/j.jpedsurg.2017.07.010 28755898

[16] Gunadi SM, Budi NYP (2018). The impact of down-regulated SK3 expressions on Hirschsprung disease. BMC Med Genet.

[17] Gunadi MK, Ohta M (2009). Two novel mutations in the ED1 gene in Japanese families with X-linked hypohidrotic ectodermal dysplasia. Pediatr Res.

[18] Gunadi, Budi N, Iskandar K, Adrianto I (2017). NRG1 rare variant effects in Hirschsprung disease patients. Ann Transl Med.

[19] Ghosh R, Oak N, Plon SE (2017). Evaluation of in silico algorithms for use with ACMG/AMP clinical variant interpretation guidelines. Genome Biol.

[20] Walters-Sen LC, Hashimoto S, Thrush DL (2015). Variability in pathogenicity prediction programs: impact on clinical diagnostics. Mol Genet Genomic Med.

[21] Yang S, Lincoln SE, Kobayashi Y (2017). Sources of discordance among germ-line variant classifications in ClinVar. Genet Med.

[22] Dong C, Wei P, Jian X (2015). Comparison and integration of deleteriousness prediction methods for nonsynonymous SNVs in whole exome sequencing studies. Hum Mol Genet.

[23] Jha P, Lu D, Xu S (2015). Natural selection and functional potentials of human noncoding elements revealed by analysis of next generation sequencing data. PLoS One.

[24] Holmes WE, Sliwkowski MX, Akita RW (1992). Identification of heregulin, a specific activator of p185erbB2. Science.

[25] Barlow A, de GVE, Pachnis V (2003). Enteric nervous system progenitors are coordinately controlled by the G protein-coupled receptor EDNRB and the receptor tyrosine kinase RET. Neuron.

[26] Paratore C, Eichenberger C, Suter U (2002). Sox10 haploinsuffciency affects maintenance of progenitor cells in a mouse model of Hirschsprung disease. Hum Mol Genet.

[27] Phusantisampan T, Sangkhathat S, Phongdara A (2012). Association of genetic polymorphisms in the RET-protooncogene and NRG1 with Hirschsprung disease in Thai patients. J Hum Genet.

[28] Kapoor A, Jiang Q, Chatterjee S (2015). Population variation in total genetic risk of Hirschsprung disease from common RET, SEMA3 and NRG1 susceptibility polymorphisms. Hum Mol Genet.

[29] Jiang M, Li C, Cao G (2017). Effects of NRG1 polymorphisms on Hirschsprung's disease susceptibility: a meta-analysis. Sci Rep.

[30] Tajima A, Pan IH, Fucharoen G (2002). Three major lineages of Asian Y chromosomes: implications for the peopling of east and Southeast Asia. Hum Genet.

