# Supplementary material for: NRG1 variant effects in patients with Hirschsprung disease
Source: BMC Pediatr. 2018 Sep 4;18:292. doi: 10.1186/s12887-018-1265-x (PMC6123906; doi:10.1186/s12887-018-1265-x)
Supplement: Supplementary file 1 — Supplementary material URLs. (DOC 22 kb) [file 12887_2018_1265_MOESM1_ESM.doc]

a) 1000 Genomes Project (http://www.internationalgenome.org); b) ExAC (http://exac.broadinstitute.org); c) SIFT (http://sift.jcvi.org/); d) PolyPhen-2 (http://genetics.bwh.harvard.edu/pph2/); e) LRT (https://www.ncbi.nlm.nih.gov/pmc/articles/PMC3910100/); f) Mutation Taster (http://www.mutationtaster.org); g) Mutation Assessor (http://mutationassessor.org/r3/); h) FATHMM (http://fathmm.biocompute.org.uk); i) CADD (http://cadd.gs.washington.edu); j) DANN (https://cbcl.ics.uci.edu/public_data/DANN/); k) GERP (http://mendel.stanford.edu/SidowLab/downloads/gerp/index.html); l) PhyloP (http://ccg.vital-it.ch/mga/hg19/phylop/phylop.html); m) SiPhy (http://portals.broadinstitute.org/genome_bio/siphy/index.html); n) ClinVar (https://www.ncbi.nlm.nih.gov/clinvar/); o) HaploReg (https://pubs.broadinstitute.org/mammals/haploreg/haploreg.php)
